# Assessment of humeral head perfusion after proximal humeral fracture - study protocol for evaluation of MR-based imaging techniques

**DOI:** 10.1186/s13018-025-05964-0

**Published:** 2025-06-03

**Authors:** Angelina Garkisch, Dagmar-C. Fischer, Thomas Mittlmeier, Sven Märdian, Bastian Klaan, Marc-André Weber, Andreas Erbersdobler, Stephan Pauly, Patrick Gahr

**Affiliations:** 1 Department of Traumatology, Hand and Reconstructive Surgery, Rostock University Medical Center, Schillingallee 35, 18057 Rostock, Germany; 2 Department of Pediatrics, Rostock University Medical Center, Rostock, Germany; 3 Institute for Diagnostic and Interventional Radiology, Pediatric and Neuroradiology, Rostock, Germany; 4 Institute of Pathology, Rostock University Medical Center, Rostock, Germany; 5 Department of Shoulder Surgery, Vivantes Auguste-Viktoria-Klinikum, Berlin, Germany

**Keywords:** Zero echo time MRI, Dynamic contrast-enhanced MRI, Proximal humeral fracture, Avascular necrosis, Humeral head, Bone perfusion

## Abstract

**Background:**

There are no clear criteria for decision-making after proximal humeral fractures, and the choice of surgical strategy and, in particular, timing is controversial. Vascular disruption can lead to delayed avascular necrosis of the humeral head, which may result in failure of the fixation procedure and the need for revision surgery. MRI pulse sequences with zero echo time (ZTE) provide “CT-like” images of the bone, while dynamic contrast-enhanced MRI (DCE MRI) allows for visualization and quantification of vascular permeability, blood flow, and clearance rates within the bone. We hypothesize that MRI with a ZTE sequence is non-inferior to CT in terms of fracture morphology specification and that DCE MRI is suitable for quantifying perfusion of the humeral head after a proximal humeral fracture.

**Methods:**

This study is designed to investigate the value of modern MRI diagnostics in proximal humeral fractures. 30 patients with an acute unilateral proximal humeral fracture and an indication for ORIF with a locking plate will receive routine diagnostics (conventional X-rays and CT), supplemented by ZTE and DCE 3 Tesla MRI protocols and clinical scores. Two follow-up examinations will be performed at 3 and 6 months after surgery.

**Discussion:**

As the optimal treatment method and timing of proximal humeral fractures are still controversial, the results of this study might help surgeons decide on the optimal treatment. The first aim is to evaluate the ZTE MRI sequence for its ability to assess fracture morphology compared to CT imaging. In this way, it can be decided whether or not the ZTE sequences can be considered as an alternative to CT imaging in proximal humeral fractures. The second aim of the study is to investigate the feasibility of DCE MRI for quantifying perfusion of the humeral head over a six-month period after proximal humeral fracture and ORIF with a locking plate. If the initial DCE MRI shows restricted perfusion of the humeral head, the risk of avascular necrosis may be increased, so it may be beneficial for the patient to proceed directly to reverse shoulder arthroplasty (RSA) instead of osteosynthesis with the risk of later revision. The data obtained from this study are required for the planning of a randomized trial.

**Trial registration:**

DRKS-ID: DRKS00031380, MRI for acute proximal humeral fractures / - Assessment of fracture geometry with an MRI application for imaging cortical bone surfaces (Part I) / - Quantification of humeral head perfusion with Dynamic Contrast-Enhanced MRI (Part II). Acronym: Rostock MRI Study. Date of Registration 2023-06-14. Recruitment is scheduled from 1st July 2023 to 31st December 2024. The final follow-up is scheduled for June 2025. https://drks.de/search/de/trial/DRKS00031380/details;jsessionid=D961FF527EB81204EB9F211810A89B73.

## Introduction

### Background and rationale

With a prevalence of 4–10%, proximal humeral fractures are among the most common fractures and are the third most frequent fractures in the elderly population [1, 2]. Thus far, conventional X-ray imaging and/or computed tomography (CT) are routinely used for the initial detection and characterization of these fractures and subsequent planning of the surgical approach, i.e. open reduction and internal fixation (ORIF) in displaced multifragmentary fracture patterns using a locking plate. However, the functional outcome is mediocre and complication rates are high, especially in elderly patients [3, 4]. Alternative approaches such as conservative therapy, intramedullary nailing, or (reverse) shoulder arthroplasty (RSA) are available, and the decision to apply either one of these techniques remains elusive [5, 6]. Despite increasing evidence that older patients suffering from complex fractures benefit from initial RSA [7], thus far no definite criteria for decision-making are available and the choice of surgical strategy is still controversial [8–10]. Furthermore, concomitant vascular disruption may lead to delayed avascular necrosis of the humeral head with subsequent failure of the fixation procedure and the need for revision surgery [11]. Thus, predicting the risk of humeral head ischemia at the time of fracture would help to reduce the number of patients at risk for revision surgery. Although Hertel et al. proposed a checklist for the evaluation of preoperative radiographs two decades ago, the predictive value of these criteria, e.g. a short dorsomedial metaphyseal extension (< 8 mm), a disruption of the medial hinge, and involvement of the anatomical neck is still controversial [12–14]. While combining all of the proposed criteria allows prediction of ischemia of the humeral head with an accuracy of up to 97%, information on the actual humeral head perfusion is not available via X-ray imaging [15]. However, if vascular necrosis actually occurs, magnetic resonance imaging (MRI) is considered the gold standard for detection [16].

While MRI is routinely chosen for the visualization of soft tissue rather than bony structures, recent developments broaden the field of application for this technique. Firstly, the possibility to apply MRI pulse sequences with ultrashort echo-time (UTE) or even zero echo-time (ZTE) enables imaging of tissues with very short T2/T2* relaxation times and low proton density like the cortical bone [17]. After post-processing, these sequences yield “CT-like” images of bones and have shown their potential value in assessing bone structures when compared to CT imaging in various locations in both, the upper and lower extremity [18–20].

Secondly, dynamic contrast-enhanced MRI (DCE MRI) enables the visualization and quantification of vessel permeability, blood flow, and clearance rates even within bone [21–26].

We hypothesize that *(i)* an MRI including a ZTE sequence is not inferior compared to CT with regard to the detection of fracture lines and specification of fracture morphology and that *(ii)* DCE MRI is suitable for quantifying humeral head perfusion after a proximal humeral fracture.

Because either one of these hypotheses is worth to be investigated within clinical studies, there is the scientific need to conduct an exploratory study first. This will not only provide the data necessary to sharpen the design of a subsequent clinical investigation but will also help to integrate the study investigations into the clinical routine.

## Methods / Design

### Aim of the study

This study aims *(i)* to evaluate a ZTE bone sequence (oZTEo, Signa Premier, General Electric Healthcare, Waukesha, Wisconsin, USA) for the assessment of fracture geometry and concomitant injuries compared to CT as the standard of care, *(ii)* to gain insights into the benefit of a pre-operative DCE MRI for prediction of humeral head perfusion, and *(iii)* to evaluate the humeral head perfusion at 3 and 6 months postoperatively. To this end, the correlation between perfusion data based on DCE MRI, the clinical (scores), and radiological findings (conventional radiographs, CT) as well as the intraoperative findings (biopsy) will be investigated. In parallel, we will compare the preoperative CT and ZTE MRI-based findings with regard to the value for fracture classification. This comparison is indispensable to assess the power of ZTE MRI as a substitute for CT imaging and thus to reduce radiation exposure. Regarding DCE MRI, patients with proximal humeral fractures may benefit from improved preoperative diagnostics and information on the perfusion of the humeral head. This could allow for an individualized decision on optimal treatment and as such may help to reduce the risk for revision surgery.

### Design and setting of the study

This is a prospective, monocentric, non-randomized study investigating the value of modern MRI diagnostics in proximal humeral fractures. The study is conducted at the Department of Traumatology, Hand and Reconstructive Surgery and the Institute of Diagnostic and Interventional Radiology, Pediatric Radiology, and Neuroradiology at Rostock University Medical Center.

Thirty patients with an acute unilateral proximal humeral fracture and a given indication for ORIF with a locking plate will receive routine diagnostics (conventional radiographs and CT), supplemented by ZTE and DCE MRI protocols, and clinical scores. Eligible patients will be informed by trained physicians and have to provide written informed consent to participate prior to any of the study examinations. Two follow-up examinations will be performed at 3 and 6 months after surgery.

### Eligibility criteria

Adults (≥ 18 years of age) presenting with an acute traumatic unilateral proximal humeral fracture of the AO subtypes 11A2.1, 11A2.2, 11A2.3, 11A3, 11B1.1, 11B1.2, 11C1.1, 11C1.3, 11C3.1, 11C3.2 and 11C3.3 and being scheduled for ORIF with a locking plate are eligible. Additional *inclusion criteria* are *(i)* the period between the fracture and the examination must be less than 21 days [27], *(ii)* the willingness to undergo serial MRI examinations, and *(iii)* the ability to give written informed consent. *Exclusion criteria* are any contraindication for MRI and/or the application of contrast-enhancing agents (e.g. claustrophobia, MRI unsafe pacemakers or other implants not compatible for MRI, metallic foreign bodies, allergies, glomerular filtration rate < 30 mL/min/1.73 m^2^ or dialysis), pregnancy or breastfeeding, pathological fractures, stress fractures, previous operations of the affected shoulder, especially with implants in situ, contraindications for ORIF, such as primary glenohumeral osteoarthritis or cuff tear arthropathy. The investigations are taken at baseline, 3 and 6 months postoperatively (Table 1).

**Table 1:** Time schedule of the study examinations

	Screening	Intraoperative	Day after surgery	3 month Follow up	6 month Follow up
Inclusion/exclusion criteria	x				
Informed consent	x				
Demographic and medical history	x				
Baseline laboratory test*	x		x	x	x
ZTE and DCE-MRI	x			x	x
X-Ray	x		x	x	x
CT	x				
NRS	x			x	x
SF36	x			x	x
EQ-5D	x			x	x
DASH	x			x	x
SSV	x			x	x
Constant Score				x	x
CCI				x	x
pathology sampling		x			

* Routinely performed serum analysis (thyroid hormones, renal function, blood cell count) plus the determination of Fetuin-A, FGF23, vitamin D and parathormone

### Sample size justification

Due to the fact that this is an explorative study on the feasibility of the imaging techniques mentioned above, sample size calculation was omitted [28]. However, a minimum sample size of 20 participants is recommended, as published elsewhere [29]. As we do not know if the results obtained via DCE MRI fit those obtained via histological examination of the biopsy we are considering investigating a total of 30 patients.

### Subjects

All subjects with a proven unilateral humeral fracture and consent to participate will receive a ZTE MRI and a DCE MRI using a clinical 3.0 Tesla MR-scanner (Signa Premier, General Electric Healthcare, Waukesha, Wisconsin, USA) in a supine position with a 30-channel Adaptive Image Receive (AIR) anterior array surface coil [23]. Patients will be examined prior to, 3 and 6 months following surgery.

### Imaging protocol

The imaging protocol consists of unilateral fat-saturated proton-density (PD) fast spin-echo (FSE) weighted sequences with the PROPELLER technique in the coronal and axial plane, unilateral T1-weighted FSE sequence in the coronal plane, unilateral 3D T1-weighted ZTE bone sequence (oZTEo) in the coronal plane, and a bilateral 3D T2-weighted short tau inversion recovery sequence (STIR) in the coronal plane. After intravenous admission of 0.1 ml Gadobutrol (Gadovist, Bayer Vital, Germany) per kilogram body weight a bilateral T1-weighted FSE DCE sequence with 7 acquisitions and a duration of 5 min as well as a unilateral fat-saturated T1-weighted FSE sequence in the coronal plane will be recorded. Per subject, the time for examination will be around 30 min. Subsequent imaging at 3 and 6 months after surgery will be the same as above plus additional unilateral T1-weighted FSE Flex-sequence and T2-weighted Multi-Acquisition with Variable Resonance Image Combination SeLective (MAVRIC SL) in the coronal plane before contrast admission. Thus, the duration of the examination is estimated at 40 min. DCE sequences will be recorded for both shoulders simultaneously to compare head perfusion of the fractured and the contralateral side. Due to the limited maximum size of the field-of-view (FoV), this will be impossible in patients with a shoulder girdle width of more than 50 cm. In these subjects, only the fractured shoulder will be imaged. The same applies to patients with prior or concomitant shoulder injuries or surgeries on the contralateral side, as a reliable comparison of blood flow is not feasible in these cases either.

### Post-processing of DCE MRI data

**Fig. 1:**
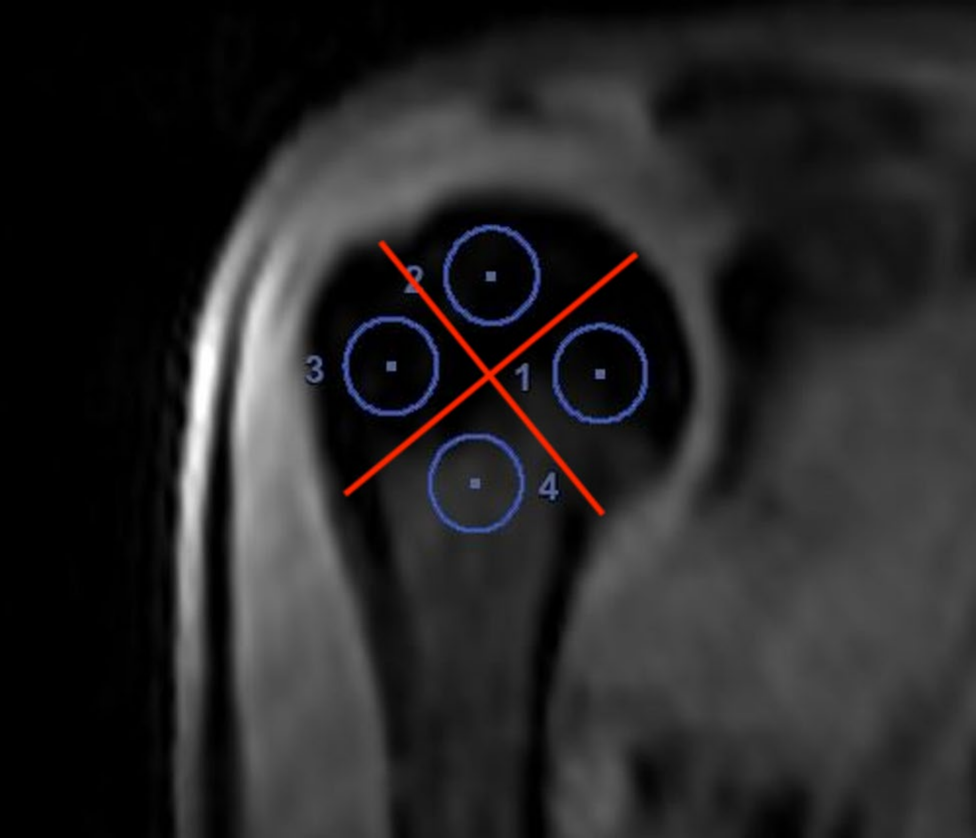
Example of positioning the 4 ROIs. Coronal view of the humeral head divided into 4 quadrants by two vertical red lines, ROIs positioned in the medial [1], superior [2], lateral [3], and inferior [4] quadrants of a right humeral head

**Fig. 2:**
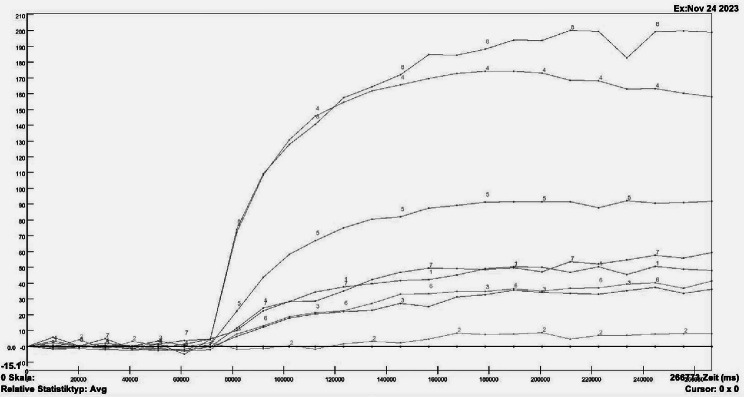
Signal intensity curves. Signal intensity curves gathered via a region of interest (ROI) analysis and subdivided depending on their contrast agent uptake. The x-axis represents the time in milliseconds, the y-axis represents the relative signal intensity in Arbitrary Units (AU)

According to Hettrich et al., within the humeral head, 4 circular regions of interest (ROIs) will be positioned in the medial, superior, lateral, and inferior quadrants as defined in the coronal plane (Fig. 1) [30]. Within each of these ROIs signal intensity curves corresponding to the uptake of the contrast agent are gathered (Fig. 2).

## Study investigations

### Preoperative

After confirming detection of a proximal humeral fracture at plain X-ray and conventional CT scans, ZTE and DCE MRI according to the study protocol will be performed.

Demographic and clinical data such as date of birth, gender, height, weight, handedness, comorbidities, time of injury, side of fracture, medication, and pain will be recorded. Pain will be quantified with a numerical rating scale (NRS; 0 = none, 10 = maximum). The Disabilities of Arm, Shoulder and Hand (DASH) score, Subjective Shoulder Value (SSV), Short Form-36 (SF-36), European Quality of Life 5 Dimensions (EQ-5D), and Charlson Comorbidity Index (CCI) will be recorded [31–36]. Blood will be sampled for pre-operative routine assessments of blood cell counts, inflammation, and coagulation features. Furthermore, we aim to investigate FGF23-, 25-Hydroxyvitamin D_3_-, PTH-, and Fetuin-A levels as established parameters for calcium and phosphate homeostasis and bone mineralization, respectively [37].

### Intraoperative

Before fracture reduction and plate insertion, a biopsy of the humeral head fragment will be taken through the fracture gap (kyphoplasty needle; gauge 09; Paragon®, Sterylab S.r.l., Italy). Biopsy results will serve as the gold standard for determining the humeral head viability according to the protocol of Larribe et al. [24]. Subsequently, open reduction and fixation with an anatomical locking plate (PHILOS, “Proximal Humerus Internal Locking System”; Synthes DePuy GmbH, Oberdorf, Switzerland) will be performed according to established routines.

### Postoperative (during hospitalization)

The expected hospital stay after operative treatment is about two days. On the morning of the first postoperative day, a blood sample will be taken for routine determination of blood loss, infection parameters, and electrolytes as well as monitoring of Fetuin-A, FGF23, Vitamin D, and PTH. As a standard of care, postoperative X-ray controls (standard two planes) of the affected shoulder will be accomplished on the second postoperative day. Follow-up examinations are scheduled at the time of discharge.

### Follow up 3 and 6 months postoperatively

In addition to the conventional X-ray imaging of the affected shoulder, patients undergo DCE MRI of both shoulders at 3 and 6 months following surgery. All of the examinations listed above (i.e. markers of bone metabolism, DASH score, SSV, CCI, SF-36, EQ-5D) together with the Constant score with myometrical testing (AFG Myometer, Mecmesin) will be performed [38]. Demographic and clinical data will be checked for completeness and the pain at rest and during efforts will be recorded with the NRS.

### Data collection, management, and statistical analysis

The demographic and clinical data at baseline and each follow-up will be gathered by interview and chart review. At enrollment, each patient receives a unique identification code (ID), and all documents and hard copies generated within this trial are linked to this identifier. The underlying key will be stored in a password-protected file accessible only to the principal investigators (AG, PG). Access to the data is limited to the researchers involved in this trial. SPSS statistical package 26.0 (SPSS Inc., Chicago, IL) will be used for statistical analyses. Normal distribution of continuous variables will be evaluated by the Kolmogorov-Smirnov test and descriptive summary measures will be computed accordingly. Categorical variables are summarized as numbers and percentages. Data from all participants will be included (intent-to-treat analysis). No missing data imputation will be performed and, given the rather small number of participants, neither subgroup nor adjusted analyses are planned.

## Discussion

The first objective is to evaluate the ZTE MRI sequence for its ability to assess fracture morphology as precisely as conventional CT imaging. This will form the basis for whether or not ZTE sequences can be considered an alternative to CT imaging in patients with proximal humeral fractures. Furthermore, the sensitivity and specificity of ZTE MRI for detecting humeral fractures will be calculated. The second study’s aim is to investigate the feasibility of DCE MRI to quantify perfusion of the humeral head over a six-month period following proximal humeral fracture treated with a locking plate. As the best treatment of proximal humeral fractures is still a matter of debate, the results of this study might help surgeons decide on the most effective treatment [6]. If the initial DCE MRI shows restricted blood flow to the humeral head, the risk of avascular necrosis may be increased, so it may be better to proceed directly to a reverse shoulder arthroplasty instead of an osteosynthesis. Finally, the power of DCE MRI to replace the needle biopsy for assessing the humeral head perfusion will be investigated. All data mentioned above are used for sample size calculation for a sound statistical investigation. Any threshold to implementing an adequately powered randomized trial will be identified, as the smooth interaction of different professionals from different departments is essential for the success of the study.

A limitation of this study is the relatively short follow-up period, which necessitates interpretation of the findings as preliminary. A longer follow-up period would be advantageous in order to detect late-onset osteonecrosis.

## Data Availability

No datasets were generated or analysed during the current study.

## Abbreviations

AFG: Advanced Force Gauge
AIR: Adaptive Image Receive
cm: centimeter
CT: Computed tomography
CCI: Charlson Comorbidity Index
DASH: Disabilities of Arm, Shoulder and Hand
DCE MRI: Dynamic contrast-enhanced magnetic resonance imaging
EQ-5D: European Quality of Life 5 Dimensions
FoV: Field-of-view
FSE: Fast spin-echo
FGF23: Fibroblast growth factor 23
MAVRIC SL: Multi-Acquisition with Variable Resonance Image Combination SeLective
MTA: Medical technical assistant
NRS: Numerical rating scale
ORIF: Open reduction internal fixation
PD: proton-density
PHILOS: Proximal Humerus Internal Locking System
ROI: Region of interest
RSA: Reverse shoulder arthroplasty
SF-36: Short Form-36
SSV: Subjective Shoulder Value
STIR: short tau inversion recovery sequence
PTH: Parathyroid hormone
UTE: Ultra-short echo time
ZTE: Zero echo time
